# End-of-life care stress in korean pediatric nurses: A cross-sectional analysis of related perceptions, attitudes, and self-efficacy

**DOI:** 10.1371/journal.pone.0340466

**Published:** 2026-01-16

**Authors:** Eul A. Shin, Eun Jung Kim

**Affiliations:** 1 Chonnam National University Hospital, Gwangju, South Korea; 2 Department of Nursing, College of Health Sciences, Honam University, Gwangju, South Korea; Nathan S Kline Institute, UNITED STATES OF AMERICA

## Abstract

Pediatric end-of-life (EOL) care imposes a significant emotional burden on nurses, often leading to elevated stress. Psychological factors, such as perception, attitude, and self-efficacy, are known to play critical roles in stress responses during EOL care in clinical settings. This study examined the effect of South Korean pediatric nurses’ perceptions, attitudes, and self-efficacy regarding EOL care on their stress levels. A cross-sectional survey was conducted involving 150 pediatric nurses working at two university hospitals in South Korea. Structured, self-administered questionnaires were used to assess their perceptions of EOL care (supportive and obstacle factors), attitudes, and self-efficacy. Data were analyzed using t-tests, ANOVA, Scheffé’s post hoc test, and Pearson’s correlation analysis. The mean pediatric EOL care stress score was 3.60 out of 5, indicating a moderate to high level of stress. Significant differences in stress were observed based on whether participants had experienced the death of a patient in their care within the previous year and whether they had received education related to EOL care. Specifically, nurses without such experiences reported significantly higher levels of stress. Correlation analysis further revealed that pediatric EOL care stress was positively associated with supportive care perception factors and obstacle perception factors, while showing a significant negative correlation with self-efficacy. No significant relationship was found between attitudes toward EOL care and stress. This study identified the major psychological and experiential factors influencing pediatric nurses’ stress related to pediatric EOL care within two tertiary hospitals in South Korea. These findings highlight the need for structured educational interventions and institutional support systems to strengthen nurses’ coping abilities and enhance the overall quality of pediatric EOL care. In particular, integrating structured EOL training into undergraduate nursing curricula and continuing professional development programs may help reduce stress, improve self-efficacy, and ultimately ensure higher-quality pediatric EOL care.

## Introduction

Death is a natural and inevitable part of life. In South Korea, 75.8% of deaths occurred in medical institutions in 2023—a 3.5% increase over the past decade—indicating a steady upward trend [1]. Notably, 81.4% of deaths among children under the age of 15 years occur in hospitals, highlighting the growing need for pediatric end-of-life (EOL) care in clinical settings [2].

Pediatric EOL care encompasses decision making regarding EOL treatment, management of physical symptoms such as pain, and communication of poor prognoses to patients’ families [3]. Given the developmental characteristics of children and the need to include families in care decisions, nurses must be able to provide individualized and family-centered care [4]. However, due to the unique emotional and ethical challenges involved in caring for dying children, pediatric EOL care is often perceived as one of the most emotionally distressing experiences in nursing practice [5]. Emotional pain, despair, guilt, and grief can persist for weeks following a child’s death, potentially leading to long-term psychological effects on nurses [6]. Many nurses report insufficient emotional support or a lack the necessary coping skills to manage these experiences [7]. In contrast to adult EOL care, the death of a child is often perceived as an “unnatural” or “unacceptable” loss, making the emotional burden even more intense. Moreover, nurses frequently witness profound parental grief and are expected to support both the child and the family, further increasing psychological strain [8]. Consequently, stress related to pediatric EOL care has been identified as a major contributing factor to job-related stress and high turnover rates [9]. It is therefore essential to recognize EOL care stress as a serious issue and equip nurses with the tools to identify and manage their symptoms proactively [10]. Developing culturally sensitive and pediatric-specific EOL education and emotional support programs is crucial to improving nurses’ coping abilities and promoting retention in this challenging clinical context.

In the Korean context, cultural norms that discourage the public expression of emotions further complicate this issue. Nurses may suppress their emotional responses out of fear of negatively impacting patients or families [11]. Moreover, barriers such as avoidance of pediatric EOL care and misconceptions about pediatric pain management hinder effective care provision [12]. Korean pediatric nurses who have experienced child deaths often report emotional difficulties, including grief and moral conflict, and are in need of greater psychological support.

Lazarus and Folkman’s stress and coping model [13] highlights that stress outcomes are shaped by how individuals appraise stressors and the resources they perceive as available for coping. In this context, understanding the facilitators and barriers to providing pediatric EOL care becomes essential, as these factors directly influence nurses’ appraisals of stressors and the coping resources they can mobilize. Nurses’ attitudes toward EOL care significantly affect the quality of care provided to patients and their families. Positive attitudes support a dignified and peaceful death, while negative attitudes may result in inadequate symptom management and uncertainty in responding to the imminent death of a child [5]. A clearer understanding of the impact of nurses’ attitudes may improve the delivery of holistic, high-quality care that fulfill physical, psychological, and spiritual needs [6].

Self-efficacy, defined as a strong belief in one’s ability to perform a role successfully, has been cited as a key motivator in clinical practice. According to Bandura’s self-efficacy theory [14], an individual’s belief in their ability to perform specific tasks strongly influences their emotional and behavioral responses. In the context of pediatric EOL care, this framework suggests that nurses with higher self-efficacy may experience less stress because they feel more competent in providing care under challenging circumstances [15]. Nurses with high self-efficacy may therefore be better equipped to support the spiritual well-being of children at the end of life and the emotional stability of their families.

Despite the importance of this issue, research on pediatric nurses’ stress related to EOL care in South Korea is limited. Previous studies have primarily focused on adult EOL care, with few examining the unique factors influencing stress among pediatric nurses [16]. Furthermore, EOL care education is not mandatory in Korea’s continuing nursing education system, and few structured programs are available [17]. In Korea, nursing education consists of either a three-year college diploma or a four-year university degree, followed by licensure through a national examination. While continuing education is mandatory for license renewal, EOL care training is not a required component [18]. Instead, it is offered as optional workshops or institution-specific programs, leading to significant variation in nurses’ preparedness. As a result, many pediatric nurses develop their EOL competencies primarily through clinical experience rather than structured training. This educational gap may exacerbate stress and hinder the delivery of high-quality pediatric EOL care.

With this in mind, this study aims to investigate the stress experienced by pediatric nurses in Korea during EOL care and to explore the impact thereof on their perceptions, attitudes, and self-efficacy. These findings are expected to provide foundational data for developing targeted interventions and educational programs to improve pediatric EOL care.

Therefore, this study addresses the following research questions:

1. What is the level of stress experienced by pediatric nurses in providing EOL care in South Korea?
2. How do perceptions, attitudes, and self-efficacy relate to pediatric nurses’ stress regarding EOL care?
3. To what extent do these factors influence the overall stress of pediatric nurses when providing EOL care?

## Methods

### Design

A cross-sectional survey design was adopted to identify the factors influencing stress related to pediatric EOL care.

### Sampling

The participants were pediatric nurses working in two tertiary general hospitals located in the Gwangju City, Jeonnam region of South Korea. Both hospitals are affiliated with the same national university and are the largest tertiary medical institutions in the region. One of these hospital is nationally recognized cancer-specialized hospital that has consistently achieved top rankings in cancer treatment quality evaluations and survival outcomes. The selection of these sites was based on both accessibility, feasibility and their high pediatric patient volume, which ensured relevance to the study purpose and facilitated participant recruitment and data collection. Convenience sampling was employed to recruit nurses who had been working in pediatric departments for more than six months and had direct experience in providing EOL care to children. Only participants who understood the purpose of the study and provided written informed consent were included. Nurses without experience in pediatric EOL care were excluded.

The required sample size was calculated using G*Power 3.1.9.7, with a significance level of.05, statistical power of 0.80, and effect size of 0.15. The minimum sample size required was 135 participants. Considering the potential dropout rate of approximately 15%, data were collected from 150 participants. All 150 responses were included in the final data analysis.

### Data collection

The data were collected between August 1 and September 30, 2024. Prior to data collection, the purpose and procedures of the study were explained to the supervisors of the participating institutions and institutional consent was obtained.

### Measurement & Instruments

#### EOL care perception.

EOL care perception was measured using an instrument originally developed by Beckstrand et al. [19] and later translated and revised by Baek and Kang, [20] with permission from the original author. Baek and Kang [20] first had a bilingual nursing professional, fluent in both English and Korean, translate the original tool into Korean. Then, another independent bilingual nursing expert performed a back-translation into English. Discrepancies between the original and back-translated versions were carefully reviewed, and the translation process was repeated until conceptual equivalence was achieved. After the translation was finalized, content validity was evaluated using the Content Validity Index (CVI) by a panel of five experts (one nursing professor and four pediatric nurses with more than 10 years of clinical experience). Based on their feedback, several items were revised to improve clarity and cultural appropriateness. The tool comprises 51 items: 32 obstacle items and 19 supportive behavior items. Each item was assessed in terms of both the perceived intensity and frequency of experience, resulting in four subscales: perceived intensity of supportive behaviors, frequency of supportive behaviors, perceived intensity of obstacles, and frequency of obstacles.

All items were rated on a 6-point Likert scale ranging from 1(“not at all”) to 6(“very frequently). Subscale scores were calculated by summing the relevant item scores, with higher values indicating stronger perceptions or more frequent experiences. Thus, a higher score on the supportive behavior subscales reflects greater recognition or frequency of supportive actions that facilitate EOL care, whereas higher obstacle subscale scores represent stronger perceptions or more frequent experiences of barriers hindering EOL care This instrument was selected because it has been widely applied in Korean nursing studies, demonstrates strong reliability, and is suitable for capturing both supportive and barrier factors that nurses encounter in EOL care.

### EOL attitudes

Attitudes toward EOL care were assessed using Frommelt’s Attitudes Toward Nursing Care of the Dying Scale [21], which was translated and modified by Cho and Kim [22] with permission from the original author. Cho and Kim [22] conducted the translation into Korean, after which two English majors performed a back-translation to verify conceptual equivalence. The translated items were then reviewed and revised based on feedback from a panel of experts consisting of two adult nursing professors and one psychiatric nursing professor, and content validity was confirmed to ensure clarity and cultural appropriateness. The instrument consists of 30 items, of which 20 measure attitudes toward dying patients and 10 measure attitudes toward the patient’s family members.

Each item is rated on a 4-point Likert scale ranging from 1(“strongly disagree”) to 4(“strongly agree”). Negatively worded items were reverse-coded prior to analysis. Subscale were calculated by summing the relevant items, and the total score was obtained by adding all 30 items. Higher scores indicate more positive attitudes toward EOL care, reflecting greater acceptance of caring for dying patients and their families. This scale was chosen because it is one of the most widely validated measures for examining nurses’ attitudes toward dying patients and their families, making it highly relevant for assessing attitudinal aspects of pediatric EOL care.

### EOL self-efficacy

EOL care self-efficacy was measured using an instrument originally developed by Pfister et al. [23] and later translated, revised, and validated by Lee [24], with the permission of the original authors. In Lee’s study, two nursing professionals translated the instrument into Korean, and content validity was evaluated by a panel of two nursing professors and three head nurses with more than 15 years of clinical experience. The translated items were then pilot-tested with a small group of pediatric nurses to ensure clarity and comprehension. Subsequently, a bilingual expert conducted a back-translation to verify conceptual equivalence, and content validity was reconfirmed in collaboration with a nursing professor. The scale comprises ten items, each rated on a 4-point Likert scale ranging from 1 (“strongly agree”) to 4 (“strongly disagree”). Negatively worded items were reverse-coded prior to analysis. The total score was calculated by summing all items. Because the majority of items are phrased negatively (e.g., “I feel uncertain about providing EOL care”), higher scores reflect greater endorsement of negative statements and therefore indicate lower levels of self-efficacy. Conversely, lower scores reflect greater confidence and competence in providing EOL care tasks. This scoring approach, although reversed compared to typical self-efficacy scales, was intentionally retained to maintain consistency with the original validated version of the instrument. To avoid confusion, the scoring direction was clearly explained in both the Methods and Discussion sections.

This scale was selected because it directly measures nurses’ confidence in delivering EOL care, a key psychological factor in understanding stress responses in clinical settings. Furthermore, it has been validated in Korean nursing populations, ensuring cultural relevance and measurement reliability.

### Pediatric EOL care stress

Pediatric EOL care stress was measured using a tool developed by Park, Ju, and Lee [25] with permission from the original author. The instrument consists of 22 items categorized into five subdomains: psychological distress (seven items), conflict with parents (five items), communication difficulties (five items), lack of EOL care knowledge (three items), and limited work environment (two items). Each item was rated on a 4-point Likert scale ranging from 1 (“not at all”) to 4 (“very much”). Subscale scores were calculated by summing the respective item scores, and a total stress score was obtained by summing all 22 items. Higher scores indicate greater levels of stress experienced by nurses when providing pediatric EOL care, reflecting stronger perceptions of psychological, communicative, or environmental challenges in this context. This instrument was selected because it was specifically developed for pediatric nursing in South Korea and captures stress factors unique to pediatric EOL care, ensuring cultural and contextual appropriateness for this study.

### Data analysis

The data were analyzed using the IBM SPSS statistical program for Windows, version 28. Descriptive statistics were used to summarize participants’ general characteristics, perceptions of EOL care, attitudes toward EOL care, self-efficacy, and stress related to pediatric EOL care.

Differences in pediatric EOL care stress according to participant characteristics were examined using independent *t*-tests and one-way ANOVA, following a test for normality. As the assumption of homogeneity of variances was satisfied, Scheffé’s test was employed for post hoc analysis, given its conservative nature in controlling Type I error across multiple comparisons. Pearson’s correlation coefficients were calculated to assess the relationships between key variables.

### Ethics statements

This study was approved by the Institutional Review Board of the Chonnam National University Hospital in South Korea (approval number: CNUHH-2024–136). Participants provided informed consent by completing a pre-consent form in the first section of the online survey. The consent form included information about the research purpose, confidentiality of personal information, and the right to withdraw without any prior justifications. The information provided was aimed at making participants aware of their rights and providing them with the opportunity to make informed decisions about participating in the study. Additionally, no personally identifiable information was collected during the online survey, and all responses were recorded anonymously. The data were stored on a password-protected server accessible only to the research team, ensuring data confidentiality and privacy throughout the study.

## Results

### Participant characteristics

A total of 150 nurses participated in the study. Their average age was 30.86 years, with 54.7% aged 29 years or younger. In terms of relationships, 72.7% of the nurses were single and 78.7% had no children. Considering education, 88.7% had a bachelor’s degree and 57.3% had no religious affiliation.

In terms of occupational characteristics, 37.3% had more than five years and less than 10 years of total work experience, and 87.3% were registered nurses. At the time of the study, they were working in the following departments: other (internal medicine ICU, surgical ICU, emergency department; 56.0%), neonatal ICU (22.7%), pediatric ICU (11.3%), and pediatric ward (10.0%). During the year prior, 66.0% had experienced five or fewer patient deaths, 80.7% had experienced no deaths, and 54.0% had received training in EOL care (Table 1).

**Table 1:** Participant characteristics (*N* = 150).

Characteristics	Categories	*n* (%)	M ± SD
Age (year)	≤ 29	82 (54.7)	30.86 ± 6.76
	30–39	48 (32.0)	
	≥ 40	20 (13.3)	
Marital status	Single	109 (72.7)	
	Married	41 (27.3)	
Child	Yes	32 (21.3)	
	No	118 (78.7)	
Education	Bachelor’s	133 (88.7)	
	≥ Master’s	17 (11.3)	
Religion	Yes	64 (42.7)	
	No	86 (57.3)	
Total career (year)	< 1	9 (6.0)	
	1– < 3	31 (20.7)	
	3– < 5	23 (15.3)	
	5– < 10	56 (37.3)	
	≥ 10	31 (20.7)	
Position	Staff nurse	131 (87.3)	
	Charge nurse	10 (6.7)	
	Head nurse	9 (6.0)	
Department	Pediatric ward	15 (10.0)	
	PICU	17 (11.3)	
	NICU	34 (22.7)	
	Other	84 (56.0)	
Patient death experience (Previous 1 year)	0	20 (13.3)	
	1–5	99 (66.0)	
	≥ 6	31 (20.7)	
Loss of family or friends (Previous 1 year)	Yes	29 (19.3)	
	No	121 (80.7)	
EOL care training experience	Yes	83 (54.0)	
	No	67 (46.0)	

*Note:* EOL = End of Life.

### Pediatric nurses’ perceptions, attitudes, self-efficacy, and stress related to pediatric EOL care

The mean score for the perceived level of supportive factors in EOL care among pediatric nurses was 10.76 (±4.86) out of a possible 25 points. The mean score for perceived obstacle factors was 8.42 (±4.00) out of 25 points.

The mean score for attitudes toward EOL care was 2.93 (±0.23) on a 4-point Likert scale, while the mean score for EOL care self-efficacy was 1.96 (±0.49), also on a 4-point scale.

The average stress during EOL care for children was 3.60 (±0.65) 5. The mean for each sub-domain was 3.77 (±0.73) for communication difficulties, 3.63 (±0.94) for restricted work environment, 3.56 (±0.70) for conflict with parents, 3.54 (±0.72) for psychological difficulties, and 3.50 (±0.90) for lack of EOL care knowledge (Table 2).

**Table 2:** Descriptive statistics for study variables (*N* = 150).

Variables/Sub-Categories	Possible range	Item range	M ± SD
EOL care perception			
Supporting factors	0–25	0–22.89	10.77 ± 4.86
Obstacles factors	0–25	0–20.80	8.42 ± 4.00
EOL care attitudes	1–4	2.33–3.57	2.93 ± 0.23
EOL care self-efficacy	1–4	1.00–3.40	1.96 ± 0.49
EOL care stress in children	1–5	1.23–4.91	3.60 ± 0.65
Psychological difficulties	1–5	1.43–5.00	3.54 ± 0.72
Conflict with parents	1–5	1.00–5.00	3.56 ± 0.70
Difficulty in communication	1–5	1.00–5.00	3.77 ± 0.73
Lack of knowledge about EOL care	1–5	1.00–5.00	3.50 ± 0.90
Limited work environment	1–5	1.00–5.00	3.63 ± 0.94

*Note*: M = Mean; SD = Standard deviation; EOL = End of Life.

### Obstacles in EOL care

Among the perceived obstacles to EOL care, “The nurse’s workload being too heavy to adequately care for the dying child” received the highest score (M = 4.30), followed by “Intra-family fighting about whether to continue or stop aggressive treatment” (M = 4.09).

Regarding the frequency of experienced obstacles, “Poor design of units which do not allow for privacy” was rated the highest (M = 3.29), followed by “Lack of nursing education regarding quality EOL care” (M = 2.88).

The top-ranked items based on magnitude scores were “Nurse’s opinion about the direction of their caring is not valued” (M = 12.97), “The nurse’s workload being too heavy to adequately care for the dying child” (M = 12.95), “Lack of nursing education regarding quality EOL care” (M = 12.54), and “Intra-family fighting about whether to continue or stop aggressive treatment” (M = 12.10). For readability, only the top 10 obstacle items are presented in Table 3, while the full ranking of all items is available in Supplementary Table 1.

**Table 3:** Rank of obstacles in EOL care(Top 10 obstacles example) (*N* = 150).

Variables	Intensity		Frequency		Magnitude	
	M ± SD	Rank	M ± SD	Rank	M ± SD	Rank
Obstacles in EOL care.	3.69 ± 0.66		2.25 ± 0.90			
Nurse’s opinion about the direction of their caring is not valued.	4.07 ± 0.98	3	3.05 ± 1.46	3	12.97 ± 7.53	1
The nurse’s workload being too heavy to adequately care for the dying child.	4.30 ± 0.81	1	2.93 ± 1.43	4	12.95 ± 7.30	2
Lack of nursing education regarding quality EOL care.	3.75 ± 1.00	12	3.18 ± 1.36	2	12.54 ± 6.93	3
Intra-family fighting about whether to continue or stop aggressive treatment.	4.09 ± 0.92	2	2.88 ± 1.41	5	12.10 ± 6.76	4
Families not ready to acknowledge their child has an incurable disease.	3.96 ± 0.83	6	2.57 ± 1.29	7	10.51 ± 6.32	5
Fear that the grieving process for the nurse will be greater if allowing themselves to become “attached” to child and family.	3.90 ± 1.04	7	2.47 ± 1.34	11	10.19 ± 6.56	6
The nurse having to deal with distraught family members.	3.72 ± 0.96	18	2.54 ± 1.45	10	9.99 ± 6.41	7
The nurse not knowing what to say to the grieving family.	3.75 ± 1.05	12	2.55 ± 1.60	9	9.90 ± 7.29	8
Lack of trust from parents in the medical system that has failed to cure their child.	3.66 ± 1.12	21	2.57 ± 1.50	7	9.82 ± 7.08	9
No available support person for the family such as a social worker or religious leader.	3.31 ± 1.22	29	2.62 ± 1.67	6	9.35 ± 7.71	10

*Note*: M = Mean; SD = Standard deviation; EOL = End of Life.

### Supportive factors in EOL care

Among the perceived supportive factors in EOL care, “allowing parents to hold the child while life support was discontinued” had the highest score (M = 4.37), followed by “having a unit schedule that allows for continuity of care for the dying child by the same nurses” (M = 4.31).

In terms of frequency of experienced supportive factors, “Having the code status of the child clearly described in the chart” was rated the highest (M = 3.54), followed by “Allowing parents to hold the child while life support is discontinued” (M = 3.40).

Regarding magnitude scores, the top-ranked items were “Having the code status of the child clearly described in the chart” (M = 15.72), “Allowing parents to hold the child while life support is discontinued” (M = 15.22), “Having a unit schedule that allows for continuity of care for the dying child by the same nurses” (M = 15.04), and “Having one family member be the designated contact person for all other family members regarding patient information” (M = 14.12). For readability, only the top 10 obstacle items are presented in Table 4, while the full ranking of all items is available in Supplementary Table 2.

**Table 4:** Rank of supportive behaviors in EOL care (*N* = 150).

Variables	Intensity		Frequency		Magnitude	
	M ± SD	Rank	M ± SD	Rank	M ± SD	Rank
Supportive behaviors in EOL care.	3.90 ± 0.74		2.70 ± 0.95			
Having the code status of the child clearly described in the chart.	4.25 ± 0.96	4	3.54 ± 1.21	1	15.72 ± 6.98	1
Allowing parents to hold the child while life support is discontinued.	4.37 ± 0.82	1	3.40 ± 1.25	2	15.22 ± 6.82	2
Having a unit schedule that allows for continuity of care for the dying child by the same nurses.	4.31 ± 0.93	2	3.39 ± 1.34	3	15.04 ± 7.43	3
Having one family member be the designated contact person for all other family members regarding patient information.	4.05 ± 1.00	9	3.31 ± 1.42	4	14.12 ± 7.72	4
Bereavement debriefing sessions to discuss how to remember/honor the child.	4.25 ± 0.91	4	3.12 ± 1.44	6	13.75 ± 7.47	5
Having family members accept that the child is dying.	4.11 ± 1.07	8	3.08 ± 1.43	8	13.42 ± 7.66	6
A unit designed so that the family has a place to go to grieve in private.	4.02 ± 0.98	10	3.12 ± 1.23	6	13.09 ± 6.61	7
Having enough time to prepare the family for the expected death of the child.	4.29 ± 0.97	3	2.94 ± 1.54	9	13.00 ± 7.62	8
Having the family physically help care for the child.	4.20 ± 0.88	6	2.93 ± 1.51	10	12.65 ± 7.61	9
The nurse having had their own previous experience with the death of a family member.	4.16 ± 1.04	7	2.80 ± 1.65	11	12.22 ± 8.27	10

*Note*: M = Mean; SD = Standard deviation; EOL = End of Life.

### Differences in EOL care stress by participant characteristics

Significant differences in pediatric EOL care stress were observed according to participants’ experiences. Approximately 81% of participants (n = 121) who had not experienced the loss of family or friends within the previous year reported significantly higher stress (M = 3.65 ± 0.63) than the 19% (n = 29) who had such experiences (M = 3.38 ± 0.70; t = –2.00, p = .048). Likewise, 46% of nurses (n = 67) without prior EOL care training reported significantly greater stress (M = 3.72 ± 0.64) compared to the 54% (n = 83) who had received training (M = 3.44 ± 0.64; t = –2.65, p = .009) (Table 5).

**Table 5:** Differences in EOL care stress by characteristics of participants (*N* = 150).

Characteristics	Categories	n (%)	M ± SD	*t* or F (*p*) post hoc^†^
Age (years)	≤29	82 (54.7)	3.59 ± 0.70	0.19 (.828)
	30–39	48 (32.0)	3.58 ± 0.58	
	≥40	20 (13.3)	3.68 ± 0.65	
Marital status	Single	109 (72.7)	3.59 ± 0.71	−0.19 (.847)
	Married	41 (27.3)	3.61 ± 0.47	
Child	Yes	32 (21.3)	3.61 ± 0.47	0.12 (.903)
	No	118 (78.7)	3.60 ± 0.70	
Education	Bachelor’s	116 (77.3)	3.60 ± 0.66	0.26 (.794)
	≥Master’s	17 (11.3)	3.56 ± 0.60	
Religion	Yes	64 (42.7)	3.63 ± 0.58	0.58 (.566)
	No	86 (57.3)	3.57 ± 0.70	
Total career (year)	<1	9 (6.0)	4.00 ± 0.80	2.19 (.073)
	1– < 3	31 (20.7)	3.68 ± 0.66	
	3– < 5	23 (15.3)	3.33 ± 0.70	
	5– < 10	56 (37.3)	3.55 ± 0.62	
	≥10	31 (20.7)	3.68 ± 0.57	
Position	Staff nurse	131 (87.3)	3.59 ± 0.66	0.09 (.912)
	Charge nurse	10 (6.7)	3.64 ± 0.53	
	Head nurse	9 (6.0)	3.68 ± 0.69	
Department	Pediatrics ward	15 (10.0)	3.83 ± 0.50	1.89 (.134)
	PICU	17 (11.3)	3.64 ± 0.55	
	NICU	34 (22.7)	3.39 ± 0.75	
	Other	84 (56.0)	3.63 ± 0.64	
Patient death experience (Previous 1 year)	0	20 (13.3)	3.68 ± 0.43	1.22 (.297)
	1–5	99 (66.0)	3.63 ± 0.67	
	≥6	26 (20.7)	3.44 ± 0.71	
Loss of family or friends (Previous 1 year)	Yes	29 (19.3)	3.38 ± 0.70	−2.00 (.048)
	No	121 (80.7)	3.65 ± 0.63	
EOL care training experience	Yes	83 (54.0)	3.44 ± 0.64	−2.65 (.009)
	No	67 (46.0)	3.72 ± 0.64	

*Note:* EOL = end of life; PICU/NICU = pediatric intensive care unit/neonatal intensive care unit; Other (MICU/SICU/ER) = medical intensive care unit/surgical intensive care unit/emergency room.

### Correlation among by variables

A correlation analysis was conducted to examine the relationships between EOL care perception (supportive and obstacle factors), attitudes toward EOL care, self-efficacy, and pediatric EOL care stress among pediatric nurses.

Pediatric EOL care stress showed a significant positive correlation with supportive care perception factors (r = .34, *p *< .001) and obstacle care perception factors (r = .35, *p *< .001) and a significant negative correlation with self-efficacy (r = –.23, *p *= .005; Table 6).

**Table 6:** Correlation among by Variables (*N* = 150).

Variables		Perceptions of EOL care		Attitudes toward EOL care	Self-efficacy in EOL care	Stress of EOL care	Psychological difficulties	Conflict with parents	Difficulty in communication	Lack of knowledge about EOL care	Limited work environment
		Supportive behaviors	Obstacles								
		r (p)	r (p)	r (p)	r (p)	r (p)	r (p)	r (p)	r (p)	r (p)	r (p)
Perceptions of EOL care	Supportive behaviors	1									
	Obstacles	.48 (<.001)	1								
Attitudes toward EOL Care		.35 (<.001)	−.04 (.652)	1							
Self-efficacy in EOL care		−.52 (<.001)	−.18 (.028)	−.36 (<.001)	1						
Stress of EOL care		.34 (<.001)	.35 (<.001)	.11 (.189)	−.23 (.005)	1					
Psychological difficulties		.38 (<.001)	.36 (<.001)	.16 (.049)	−.35 (<.001)	.87 (<.001)	1				
Conflict with parents		.33 (<.001)	.35 (<.001)	.24 (.003)	−.15 (.063)	.78 (<.001)	.56 (<.001)	1			
Difficulty in communication		.20 (.017)	.33 (<.001)	.05 (.555)	−.15 (.073)	.88 (<.001)	.70 (<.001)	.60 (<.001)	1		
Lack of knowledge about EOL care		.24 (.004)	.34 (<.001)	.07 (.398)	−.07 (.422)	.82 (<.001)	.61 (<.001)	.56 (<.001)	.68 (<.001)	1	
Limited work environment		.20 (.015)	.33 (<.001)	.04 (.624)	−.18 (.033)	.81 (<.001)	.62 (<.001)	.54 (<.001)	.71 (<.001)	.71 (<.001)	1

*Note:* EOL = end of life.

Correlation analyses between pediatric EOL care stress subdomains and key psychological variables are presented in Table 6. All five pediatric EOL care stress subdomains—psychological difficulties, conflict with parents, difficulty in communication, lack of knowledge about EOL care, and limited work environment—showed significant positive correlations with EOL care perception (supportive and obstacle factors).

In contrast, attitudes toward EOL care demonstrated a significant positive correlation only with the conflict with parents domain (r = .24, p = .003). Regarding self-efficacy, significant negative correlations were observed in the domains of psychological difficulties (r = –.35, p < .001) and limited work environment (r = –.18, p = .033).

## Discussion

This study examined the level of stress experienced during pediatric EOL care by pediatric nurses working in two tertiary hospitals in South Korea, and explored its influencing factors. The average pediatric EOL care stress score among participants was 3.60 out of 5, indicating a moderate to elevated level of stress. This finding is consistent with those of the previous studies conducted among NICU and pediatric nurses [26]. Among the subdomains, the highest stressor was “communication difficulties,” followed by “limited work environment,” “conflict with parents,” “psychological distress,” and “lack of knowledge.” This aligns with previous findings from both South Korea and Turkey, where nurses reported communication—especially with pediatric patients and their families—as the most challenging aspect of EOL care [27]. Communication challenges may arise because of the developmental stages of children, emotional strain of parents, and discomfort surrounding death-related conversations. However, existing studies report that families consistently express the need for clear, honest, and compassionate communication [28]. Despite this, nurses often lack training and experience in delivering bad news and navigating conversations about serious illness [29] even though they serve as frontline communicators in multidisciplinary teams [30]. Thus, communication-focused education tailored to pediatric EOL settings should be integrated into undergraduate nursing curricula and continuing professional development.

The highest-rated barrier to care perception was “the nurse’s opinion about the direction of care is not valued,” which highlights findings from previous studies that reveal a lack of professional recognition as a key barrier to EOL care [30]. Although nurses play a critical role in advocacy and mediation between families and physicians [31], hierarchical relationships in Korean healthcare settings often limit nurses’ involvement in decision making [32]. Hospitals must therefore foster a more inclusive culture that values nurses’ input through open communication and shared decision-making environments.

The second-highest barrier was “the nurse’s workload being too heavy to adequately care for the dying child.” This is reflective of the high nurse turnover rates in Korea due to excessive workload and poor working conditions [33]. Previous studies also indicate that increased administrative burden after a child’s death intensifies EOL care stress [34]. The third-highest barrier was “lack of nursing education regarding quality EOL care.” In Korea, EOL care is not a required component of undergraduate nursing programs [35]. Nurses who frequently encounter death require structured and standardized EOL education to effectively manage care and reduce stress.

Conversely, the highest-rated supportive factor was “having the code status of the child clearly described in the chart.” This reflects the importance of clear documentation to guide decision making during emotionally and ethically complex situations—such as the continuation or withdrawal of life-sustaining treatment [36]. Clear code status reduces ambiguity and the psychological burden for nurses.

The second most supportive factor was “allowing parents to hold the child while life support is discontinued.” This is in line with existing findings that emphasize the importance of supporting family grieving processes through compassionate care [37]. Emotional support and education are essential to help nurses empathize with families during child loss.

The third most important supportive factor was “having a unit schedule that allows for continuity of care for the dying child by the same nurses.” Korean nurses often develop a strong rapport with patients and their families. Previous research highlights that therapeutic relationships built on continuity and mutual trust are crucial in providing holistic EOL care [38]. Regarding participant characteristics, stress was significantly higher among nurses with no personal experiences of death within the previous year. While this differs from some NICU-focused studies [39], it aligns with the finding that hospice nurses with such experiences show more positive attitudes toward EOL care [40,41]. Familiarity with death may enhance their understanding and acceptance, leading to reduced stress.

Consistent with our findings, nurses without prior EOL care training reported significantly higher stress, whereas those with training reported lower stress levels. This supports previous evidence that higher clinical competence and education are associated with reduced stress among nurses [42]. Moreover, recent studies reinforce the value of structured EOL education programs. Abuhammad et al. [43] found that targeted interventions enhanced NICU nurses’ competencies and reduced stress in neonatal palliative care contexts. These results underscore the importance of implementing accessible, skills-based EOL care education to enhance nurses’ preparedness and resilience, ultimately improving the quality of pediatric EOL care.

This study also found significant positive correlations between EOL care stress and both supportive behavior and barrier perceptions, suggesting that heightened awareness—whether of supportive or obstructive factors—can increase perceived stress. Notably, in this study, higher recognition of supportive factors was associated with greater EOL care stress. While supportive elements are generally expected to reduce stress, paradoxically, heightened awareness of being supported may amplify nurses’ recognition of the gravity of EOL care, thereby increasing their emotional burden. Park and Jeong [8] similarly reported that compassionate competence, a supportive factor, was positively associated with EOL care stress among pediatric nurses. Furthermore, Kim and Choi [44] found that among novice nurses, higher levels of empathetic capacity—a component of supportive factors—were linked to greater EOL care stress. These findings indicate that supportive factors may not only buffer stress but can also intensify nurses’ cognitive and emotional burden in the context of EOL care.

A significant negative correlation with self-efficacy highlights the role of confidence in reducing stress, supporting previous literature that emphasizes facilitators and barriers to delivering EOL care [45]. Because this scale consists of negatively worded items, higher scores indicate lower self-efficacy (i.e., greater uncertainty or lack of competence), whereas lower scores reflect greater confidence in providing EOL care. Interestingly, no significant correlations were observed between EOL care attitudes and stress. This contrasts with studies on adult care nurses, but aligns with previous research focused on pediatric nurses in tertiary hospitals [46]. The relationship between EOL attitudes and stress may differ according to patient population and institutional setting. One possible explanation is cultural: in South Korea, nurses often provide care within hierarchical healthcare environments, where their attitudes may have less direct influence on decision-making processes, thereby weakening the observable link between attitudes and stress. In addition, Korean cultural norms emphasize emotional restraint, respect for authority, and group harmony. Within this context, nurses often learn and conform to organizational “unspoken rules” and adapt to the hierarchy through silence and compliance in order to maintain team cohesion [47]. This process can lead them to internalize rather than express their stress, thereby increasing psychological burden during EOL care. Such emotional suppression may not only make the influence of attitudes less observable but also result in underreporting of stress levels.

Another explanation may be methodological: attitudes were measured through self-reported questionnaires, which are susceptible to social desirability bias, potentially masking the true relationship between attitudes and stress. Nurses may have presented themselves as professional, compassionate, or accepting of EOL care, even if they experienced discomfort or difficulty. This tendency may have diluted the true relationship between attitudes and stress.

These factors suggest a need for more targeted and repeated studies in pediatric contexts to disentangle cultural and methodological influences [27]. In particular, qualitative or mixed-method approaches may provide deeper insight into how cultural expectations shape nurses’ emotional experiences and coping strategies.

Based on the findings of this study, healthcare institutions should establish policies that formally recognize the critical role of nurses in pediatric EOL care. These policies should include appropriate staffing adjustments that reflect the increased workload associated with caring for patients at the end of their life, as well as the development of effective interdisciplinary communication systems. Furthermore, it is essential to integrate specialized educational programs on EOL care and family support into undergraduate nursing curricula and continuing professional development. Such efforts could enhance nurses’ attitudes and self-efficacy regarding EOL care, reduce their stress levels, and ultimately improve the quality of pediatric EOL care.

### Limitations and strengths

This study has several limitations. First, the research sites were restricted to two tertiary hospitals in the Gwangju city, Jeonnam region, chosen primarily for accessibility and feasibility by the research team. As a result, the findings may not fully represent the experiences of pediatric nurses in other regions of South Korea. Future research should include more diverse geographic areas and healthcare institutions to enhance the generalizability of the results. Second, as convenience sampling was employed, there is a possibility of self-selection bias. Nurses who were more confident or comfortable with EOL care may have been more likely to participate, which could have led to an overestimation of positive perceptions, attitudes, or self-efficacy. Future studies should consider random sampling or a more diverse participant pool to minimize this limitation. Third, the data were obtained through a self-administered questionnaire comprising numerous items, which may have limited the accuracy with which the participants expressed their emotional experiences. In addition, potential response biases, including self-report biases and social desirability bias (the tendency to provide responses perceived as favorable), may have influenced the results. Fourth, because this study employed a cross-sectional design, causal relationships between perceptions, attitudes, self-efficacy, and stress cannot be determined. To better clarify these relationships, future longitudinal or interventional studies—such as structured EOL education programs—are needed to establish causal pathways and to evaluate how changes in EOL care competencies influence stress and self-efficacy. Furthermore, future research should incorporate multivariate or more advanced analytical approaches to examine potential mediators and confounding factors that were beyond the scope of the present study. In addition, qualitative or mixed-method studies will be necessary to more comprehensively explore the cultural and institutional factors that shape pediatric nurses’ experiences with EOL care. Fifth, the findings of this study are situated within the cultural and institutional context of South Korea, which may limit their generalizability to other countries with different healthcare systems or cultural norms related to EOL care. Finally, since data collection was conducted over a relatively short two-month period, seasonal or situational factors (e.g., workload variation, patient volume, or institutional schedules) may have influenced participants’ reported stress levels. Future studies should extend the data collection period to capture potential temporal variations and reduce short-term contextual influences.

Despite these limitations, this study has several notable strengths. First, it is the first study in South Korea to comprehensively examine how pediatric nurses’ perceptions, attitudes, and self-efficacy collectively influence stress related to pediatric end-of-life (EOL) care. This contributes to a deeper and theory-informed understanding of the multidimensional nature of EOL care stress. Second, the study included pediatric nurses across a broad range of clinical environments—including general pediatric wards, pediatric intensive care units, neonatal intensive care units, emergency departments, and adult ICUs where pediatric EOL care occasionally occurs. This diverse sampling enhances the real-world applicability of the findings and reflects the complexity of pediatric EOL care across multiple clinical settings. Third, by assessing both supportive and obstructive perception factors related to EOL care, the study provides nuanced insights that may inform practical strategies to strengthen facilitators and mitigate barriers in pediatric EOL care environments. Fourth, incorporating variables such as previous EOL care training and personal experiences with patient deaths offers evidence directly applicable to workforce development, professional education, and institutional policy decisions. These findings may guide the development of more targeted EOL education programs and improvements in staffing models. Fifth, the study identifies essential competencies required for high-quality pediatric EOL care—such as communication, emotional regulation, and ethical decision-making—providing foundational data for the creation of standardized, competency-based pediatric EOL education curricula.

Finally, by reflecting the cultural and organizational characteristics of the Korean healthcare system, this study provides important context-specific insights into how hierarchical structures and norms of emotional restraint shape the stress experienced by pediatric nurses. These culturally grounded findings allow for meaningful cross-cultural comparisons and inform the development of interventions tailored to diverse healthcare environments. Therefore, the data from this study may serve as a meaningful foundation for developing specialized educational and organizational strategies to support pediatric nurses in delivering high-quality EOL care.

## Conclusions

The findings of this study suggest that, among the various factors examined, barrier and supportive behavior perceptions regarding EOL care were the most significant predictors of stress among pediatric nurses. Additionally, experiences of death within the previous year and participation in EOL care education were negatively associated with pediatric EOL care stress. These results highlight the need to reduce the perceived barriers and enhance support systems in a manner that is practically applicable to clinical settings. Moreover, there is a pressing need to improve the quality of EOL care education and develop supportive strategies that help nurses utilize their experiences of patient death as positive resources for personal and professional growth.

Given the unique demands of pediatric EOL care, a multifaceted approach is required to reduce stress among pediatric nurses and enhance the quality of care provided to dying children and their families. Tailored interventions that address both psychological and practical challenges in EOL care may ultimately improve the well-being of nurses and the dignity of care at the end of life.

## Supporting information

S1 File Raw data and supplementary tables used in the analysis of end-of-life (EOL) care stress. (XLSX)
